# Islamic perspectives on preconception, prenatal, and perinatal counseling

**DOI:** 10.3389/fped.2024.1373918

**Published:** 2024-02-21

**Authors:** Abdullah Bin Shoaib

**Affiliations:** ^1^ Department of Pediatrics, University of Texas Southwestern Medical Center, Dallas, TX, United States; ^2^ Division of Neurology, Children’s Medical Center, Dallas, TX, United States

**Keywords:** Islam, Muslim, bioethics, abortion, prenatal, perinatal, genetic, counseling

## Abstract

With advances in prenatal imaging, genetic testing, and medical treatment of premature neonates, new bioethical challenges have arisen. Many Muslims turn to their faith and religious leaders to help navigate these novel challenges. This article briefly discusses the factors that are involved in religious leaders issuing a fatwa, or religious opinion. Using clinical scenarios, this article reviews the current discourse amongst Islamic scholars and laws in Muslim-majority countries regarding challenging bioethical topics surrounding preconception counseling, antenatal testing and termination of pregnancy, as well as Islamic scholars’ attempts to determine a minimum gestational age of viability using primary religious texts, the Quran and hadith. Challenges and shortcomings in the Islamic perspective on these issues are also addressed to highlight areas in which further research should be pursued. A deeper understanding of Islamic religious perspectives on these topics can help clinicians in providing care that is informed by patients’ cultural and religious values.

## Introduction

Recent advances in medical technology have led to an increase in the frequency of prenatally diagnosed disorders which can carry uncertain or potentially life-limiting prognoses. With these advances in prenatal imaging, genetic testing, and medical treatment of premature neonates, new bioethical dilemmas have arisen (1). Faced with moral dilemmas, people may turn to culture and religion for guidance. Many Muslims turn to their religious tradition and leaders for help in navigating through these novel challenges. With large populations in the Middle East, Northern Africa, and South Asia, and significant minority populations in Europe and North America, Muslims comprise nearly a quarter of the world's population (2). Understanding the fundamentals that guide Islamic law, or *shariah*, can shed light on how certain religious rulings surrounding contemporary bioethics have been formulated. As clinicians, it is important to understand the values and beliefs that shape patients’ decisions so as to better provide care that is congruent with their value system.

*Shariah* is based on two main primary religious sources—the Quran, which Muslims believe is the immutable word of God, and the actions and quotes of the Prophet Muhammad, whom Muslims believe to be the final messenger of God, collected in books called *hadith* (3). With these primary sources, Islamic religious scholars, derive rulings, also being factoring information from the ‘*aql* (intellect) and *‘urf* (cultural considerations) (4). When Islamic scholars are tasked with deriving a religious opinion, or *fatwa* (pl. *fatawa*) on a matter, they consider these primary sources and secondary considerations in deriving a ruling consistent with the goals of *shariah*. Some of these goals of *shariah* include preserving life, avoiding or minimizing harm, and preserving *karamah*, or human dignity (3–5). In the modern era, *fatawa* are issued by prominent Islamic scholars or by groups of Islamic scholars, and can be published in books, magazines, or online (6, 7). *Fatawa* are by definition nonbinding, but in certain Muslim-majority countries, such as Saudi Arabia, *fatawa* issued by state-led panels are a basis of legislation (7–9).

This review article discusses some of the novel bioethical challenges faced when counseling patients through the preconception, prenatal, and perinatal stages. We present the case of a patient through these three stages, and the Islamic perspective on bioethical dilemmas that may arise in counseling patients through these stages.

## Case 1: preconception counseling in the Muslim world

*A Muslim woman, who emigrated from Jordan, presents to an obstetric clinic to establish care after a positive pregnancy test. She is joined by her husband, who is also her first cousin. The couple expresses that there is a strong family history of thalassemia, and are afraid that their children may be afflicted by thalassemia. What is the role and permissibility of prenatal genetic testing from an Islamic perspective?*

A sizeable portion of marriages in the Muslim world, including countries in the Middle East, North Africa, and South Asia, are consanguineous, with intrafamilial marriages accounting for close to half of all marriages in certain regions (10, 11). The factors that lead to this high rate of consanguinity are complex and multifactorial, and include educational level, socioeconomic status, and religion (10–12) With increasing rates of consanguinity, the risk of children with certain health conditions, particularly autosomal recessive conditions, increases significantly (10, 13). Therefore, it is important to ask couples about any history of consanguinity, especially particularly if they are from regions of the world where there is a high incidence of intrafamilial marriage.

Given the risks associated with certain autosomal recessive diseases, and the increased risk of bearing children with these diseases due to the frequency of consanguinity, many Muslim-majority countries have begun premarital screening for genetic conditions. For instance, in 2004, Saudi Arabia began mandatory screening tests for all couples applying for a marriage license (14–16). This test was aimed at screening for sickle cell disease and thalassemia. If the couple was found to be low risk, they would be granted a marriage license. However, if both members of the potential couple were found to have sickle cell trait or thalassemia trait, they would not receive a marriage license until after they met with a genetic counselor. The genetic counselor would then discuss the risk of certain conditions being passed on to their children (14). Couples had the right to move forward with marriage regardless of the results of this screening. Though this attempt was successful in identifying at-risk couples and providing them information from a genetic counselor, nearly 90% of couples married each other despite being aware of the increased risk of their child being affected by sickle cell disease or thalassemia (14). Other Muslim-majority countries have implemented similar programs, such as Qatar, the United Arab Emirates, Bahrain, Jordan, and Maldives (17–20).

These programs had been met with some hesitation. Besides educational and socioeconomic factors making it difficult to pursue genetic testing in certain regions, there also seems to be some hesitancy that is rooted in religious and cultural beliefs, as well as financial constraints (14, 15, 21). Some reasons cited in small cohort studies suggest that families had reservations towards genetic services because they felt that genetic counselors would be dismissive of their religious beliefs or that the conversations with genetic counselors would be directive in nature as opposed to purely informative (21, 22). Some of this hesitation is shaped in part by lack of understanding, as families with a better understanding of genetics tend to have a more positive attitude towards genetic tools (23).

From a theological perspective, Islamic organizations from majority Muslim countries have endorsed the permissibility of prenatal genetic testing from a religious perspective as prenatal genetic testing is often low-risk to the parents or the fetus, and is congruent with the principle of *shariah* to minimize harm (14, 17–20) However, in more invasive genetic testing, such as amniocentesis or chorionic villi sampling, which bears a higher risk to the fetus, the risks of the procedure must be weighed by the possible benefit gained by obtaining further information.

Although multiple religious leaders have permitted prenatal genetic testing, cultural and personal factors may also play a significant role in acceptance of these technologies by patients (22, 24). Though genetic counseling training programs have started to develop in the Muslim world, further development of training programs that delve into the unique cultural and religious beliefs of Muslims can help in guiding patients through these challenging situations in an informative, nonjudgmental and nonprescriptive manner (25).

## Case 2: Islamic perspectives on termination of pregnancy and preimplantation genetic testing (PGT)

*The couple moves forward with carrier screening for thalassemia, which is negative. The pregnancy proceeds without complications until the 21st week, at which point an anatomy ultrasound is performed and reveals an encephalocele. Fetal magnetic resonance imagining (MRI) confirms presence of a massive occipital encephalocele containing cerebral tissue, with herniation of the brainstem and cerebellar tissue. In consultations with Maternal Fetal Medicine, Neurology, Neurosurgery, Neonatology, and Palliative Care, the option of termination of pregnancy was discussed with family. What is the Islamic perspective on termination of pregnancy?*

When faced with a severe or life-limiting prenatal diagnosis, one option that families may consider is termination of pregnancy. Studies suggest that Muslims may be less likely to be agreeable to termination of pregnancy than other religious groups (24, 26, 27).

The Islamic perspective on termination of pregnancy is a somewhat nuanced, and requires understanding of the key principles of *shariah* to fully understand and appreciate. There is a *hadith* that divides embryonic development into two stages—before “ensoulment”, or the process of the soul entering the fetus, and after ensoulment. The pre-ensoulment period is further divided into three periods lasting 40 days. Though some Islamic scholars view these three periods as occurring concurrently, and thus state that ensoulment occurs by 40 days after fertilization, the majority opinion is that ensoulment occurs at 120 days after fertilization, or approximately 19 weeks gestation. Before ensoulment, termination of pregnancy is permissible in certain circumstances, as termination of pregnancy is not tantamount to taking of a life or soul (15, 25, 28). This is exemplified by a *fatwa* issued in 2011 by Saudi Arabia's Standing Committee for Scientific Research and for Issuing Edicts stating that termination of pregnancy is permissible before 120 days of pregnancy in situations in which there are serious fetal anomalies or a diagnosis that would cause severe incurable disabilities (28). Similarly, another *fatwa* issued by the International Islamic *Fiqh* Council (IIFC), an international consortium of Islamic scholars, states that termination of pregnancy is permissible before 120 days of pregnancy in the presence of severe fetal anomalies (28). Noteworthy is that both of these *fatawa* explicitly mention that multiple physicians must be consulted and be in agreement to the presence of severe fetal anomalies, highlighting the importance that these *fatawa* place in the physicians’ expertise and intellect, or ‘*aql* (28). Similar *fatawa* have been issued in Egypt, Kuwait, Pakistan, Tunisia, and Iran (which, unlike the aforementioned majority *Sunni* countries, is a majority *Shi’ite* country) (25, 28–30). There have been attempts by Islamic scholars to compile a list of fetal anomalies that would be severe enough to permit termination of pregnancy and includes conditions such as trisomy 13, trisomy 18, and bilateral renal agenesis, among other conditions (8). Though this is the majority opinion amongst Islamic scholars, there is a sizeable minority that state that termination of pregnancy is impermissible, even before 120 days (25). However, termination of pregnancy is permitted at any gestational age if pregnancy puts the mother's life at risk (15, 28).

Studies have found that a key factor in Muslims’ opposition to termination of pregnancy is based in religion, and that higher degrees of religious adherence are associated with less acceptance of termination of pregnancy (26, 27, 31). Aside from religion, acculturation also plays a significant role in views towards termination of pregnancy (24, 31). The severity of the condition that the fetus is diagnosed with may also play a role in how accepting families may be of termination of pregnancy (24). Even though families may believe that Islam does not permit termination of pregnancy, they themselves may find it personally acceptable based on their own personal experiences (32). In these situations, Muslim families may feel that they are faced with two competing interests—to do what they feel is most consistent with their religious beliefs versus to do what they personally feel is in their fetus’ best interest (15). Speaking to an *imam* and educating families about the *fatawa* permitting termination of pregnancy in certain circumstances has changed attitude towards permissibility of termination of pregnancy, and may help families reconcile what families may view as seemingly opposing views (15, 26, 33). At the same time, clinicians should be cognizant of the fact that despite the fact that Muslim families may be aware of the Islamic restrictions, and in certain cases, prohibition, on termination of pregnancy, that may be the option families decide to proceed with (31–33).

Another novel technology which has raised further ethical questions is the rise of *in vitro* fertilization (IVF) with preimplantation genetic testing (PGT). In couples undergoing IVF, and with a history of being carriers for autosomal recessive conditions, embryos can be examined and those which are confirmed to not carry the inherited genetic defect can be selected (34). This has been deemed permissible from an Islamic perspective, as this process occurs well before ensoulment, which occurs 120 days post-fertilization, and can be a consideration for some Muslim families who have a history of inherited disorders (18, 25). However, this procedure can be cost-prohibitive, and may be more difficult to obtain in parts of the developing world (25).

With these considerations in mind, there are challenges that clinicians may face. First and foremost, certain fetal anomalies are not detected until after the window of permissibility of termination of pregnancy. Guidelines by the American College of Gynecology (ACOG) recommend a standard anatomy ultrasound examination between 18 and 22 weeks gestation for low-risk pregnancies (35). However, this examination may take place after the time of ensoulment, after which point Muslim families may struggle with termination of pregnancy if a severe fetal malformation is found (28, 35). One potential means to mitigate this conflict is to attempt to perform the standard second trimester ultrasound at 18 weeks, and pursue any follow-up imaging, such as fetal MRI, promptly. However, logistically, this may be challenging, especially in areas with sparse specialty medical care. ACOG guidelines recommend earlier and more frequent ultrasound examinations in cases where there is an increased risk of fetal malformations, which would be helpful in mothers who had a prior pregnancy with a severe fetal malformation, but for a first-time mother with no family history, earlier anatomy scans would not be performed (35).

In addition, the referenced *fatawa* emphasize the importance of physicians’ input on the prognosis of the fetus (28, 29). However, there is still a great deal of uncertainty regarding certain diagnoses (1). Even from center to center, outcomes for certain diagnoses can vary and can be very dependent on the resources available, especially in developing countries. Though giving physicians latitude to guide patients on a case-by-case basis can be helpful in providing care tailored to the family's unique circumstances, it can also lead to what families may perceive as inconsistencies between clinicians’ recommendations.

## Case 3: post-natal dilemmas and Islamic perspectives on minimum age of viability

*The family elects to continue the pregnancy and at 23 weeks, the mother goes into preterm labor. Infant is apneic and bradycardic in the delivery room, but stabilized after intubation. What is the Islamic perspective on withholding and withdrawing life-prolonging treatments for neonates? Have Islamic scholars come forth with a minimum age of viability?*

Multiple Islamic organizations and scholars have permitted withholding and withdrawing of life-sustaining treatments (4–6, 36–40). Though much of the literature has been focused primarily on adults, the key principles allowing withholding and withdrawal of life-sustaining treatment is applicable to neonates and children as well (4). In summary, Islamic scholars state that seeking medical treatment is only mandatory if forgoing treatment will lead to loss of life or limb, and if the treatment will nearly certainly prevent this from happening, such as the use of antibiotics in treating a pneumonia (5, 9). However, with advances in medical technology, there is much more ambiguity in the risks and potential benefits of certain treatments. Islamic scholars state that the importance of preserving life must also be weighed against the importance of preserving *karamah*, or human dignity (5). As such, the benefits of preserving life by pursuing treatments such as tracheostomy or gastrostomy tube placement may be outweighed by the harms to the patient's dignity and quality of life (5). Though there have been attempts to establish an acceptable threshold of an acceptable quality of life based on Islamic principles, this has been unsuccessful and will vary from family to family (5, 41, 42). In neonates, this ethical challenge is further complicated by the fact that it can be quite challenging to predict the long-term outcome in rare conditions, or when the diagnosis still pending workup with genetic or other specialized testing (4).

With the infant being born at 23 weeks gestation, this case also highlights another novel challenge posed by physicians in the Muslim world—what is the minimum age of viability? Al-Alaiyan first suggested a national lower limit of viability for Saudi Arabia of 25 weeks gestation (7). This gestational age was based on scientific evidence and expert intellect (or ‘*aql*) demonstrating poor outcomes in neonates born significantly premature (7, 40, 43). In addition, he cites primary religious texts, in this case, verses of the Quran, which he states supports a minimum age of viability of roughly 6 lunar months (7). This opinion was echoed by a *fatwa* issued in 2008 by the General Presidency of Scholarly Research and *Ifta* in Saudi Arabia stating that if two physicians may withhold resuscitation if a neonate was born prior to 6 lunar months, or 25 weeks (8). However, this *fatwa* is not based on any explicit instruction from any primary religious text, and also seems to be shaped by published outcomes for premature infants in 2008, and by the landscape of healthcare resources in Saudi Arabia in 2008 (4). This raises the question of whether or not this *fatwa* would apply outside of Saudi Arabia, especially as outcomes improve for premature infants born at 23 and 24 weeks with improvements in medical care (4). This same conflict and difference of opinion is reflected in American Muslim physicians practicing (44). No other *fatawa* discussing this topic have been issued, further illustrating the lack of scholarly consensus on this matter.

## Conclusion

With advances in medical technology, new ethical challenges have arisen. Islamic scholars, using primary religious sources, the Quran and *hadith*, have offered religious opinions, or *fatawa*, addressing these issues (3, 5, 39). These opinions rely on primary religious sources, but are also shaped by the intellect, or ‘*aql*, or of medical professionals, and by the emphasis Islamic law, or *shariah*, places on minimizing harm, preserving life and preserving human dignity, or *karamah* (3). At times, the preservation of human dignity and human life may be at odds, and it is these competing interests that shape the basis of the opinions present in the literature. For instance, the permissibility of termination of pregnancy before 120 days after conception in cases of severe fetal malformations is shaped by *hadith*, but also by the emphasis on preservation of human dignity (8, 25, 28, 29). It is this same conflict that shapes the basis of the permissibility of withholding or withdrawal of life-sustaining treatments in neonates, and what has led some Islamic scholars to opine on a minimum age of viability, as well as what an acceptable “quality of life” would constitute from a religious perspective (4, 41, 42). The lack of a centralized clergy in Islam, as well as the various factors involved in developing a *fatwa* can lead to a variety of different opinions, and families may opt to follow a *fatwa* inconsistent with the *fatawa* discussed here for a variety of reasons (4). Even if families acknowledge and believe that certain actions are deemed impermissible from an Islamic perspective, they may find certain decisions personally acceptable (32).

With further advances in medical technology, more advanced diagnostics, and improved outcomes for prenatally diagnosed conditions, *fatawa* may change, though the underlying principles shaping the formulation thereof will remain consistent. Further collaboration between medical professionals, Islamic religious scholars, and chaplains is vital in furthering this area of research. Understanding the factors that shape patients’ values allows healthcare professionals to provide clinical care of higher quality that is free from prejudice and congruent with patients’ religious beliefs, culture, and value system.
